# Distinct Lineage of Vesiculovirus from Big Brown Bats, United States

**DOI:** 10.3201/eid1912.121506

**Published:** 2013-12

**Authors:** Terry Fei Fan Ng, Cindy Driscoll, Maria Paz Carlos, Algernon Prioleau, Robert Schmieder, Bhakti Dwivedi, Jakk Wong, Yunhee Cha, Steven Head, Mya Breitbart, Eric Delwart

**Affiliations:** Blood Systems Research Institute, San Francisco, California, USA (T.F.F. Ng, J. Wong, Y. Cha, E. Delwart);; University of California, San Francisco (T.F.F. Ng, E. Delwart);; University of South Florida, St. Petersburg, Florida, USA (T.F.F. Ng, B. Dwivedi, M. Breitbart);; Maryland Department of Natural Resources, Oxford, Maryland, USA (C. Driscoll);; Maryland Department of Health and Mental Hygiene, Baltimore, Maryland, USA (M.P. Carlos, A. Prioleau);; San Diego State University, San Diego, California, USA (R. Schmieder);; The Scripps Research Institute, La Jolla, California, USA (S. Head)

**Keywords:** Rhabdoviridae, vesiculovirus, viral metagenomic, bats, virus discovery, big brown bats, viruses, North America, Maryland, United States, rabies, Eptesicus fuscus

## Abstract

We identified a novel rhabdovirus, American bat vesiculovirus, from postmortem tissue samples from 120 rabies-negative big brown bats with a history of human contact. Five percent of the tested bats were infected with this virus. The extent of zoonotic exposure and possible health effects in humans from this virus are unknown.

Bats are reservoirs for many emerging viral pathogens, including Ebola viruses, Marburg viruses, henipaviruses, and severe acute respiratory syndrome coronaviruses; >80 bat virus species have been characterized (*1*,*2*). The diversity of these viruses and their high infection rates in bats may be attributed to multiple factors might that facilitate virus transmission and maintenance, including bats’ large social group size, high species diversity, long life, long-distance migration, roost sharing by multiple species, and social habits such as mutual grooming and biting (*1*,*2*).

Rabies virus (family *Rhabdoviridae*, genus *Lyssavirus*) is commonly detected in bats from the United States. Analyses of several cases of human rabies infections have reported insectivorous bats as the source (*3*). The *Rhabdoviridae* family contains 6 formally approved genera, but most bat rhabdoviruses belong to the *Lyssavirus* genus (Figure). Nonrabies lyssaviruses have been characterized from bats in other parts of the world, including Australia, Europe, Africa, and Asia (*4*–*9*). In contrast to the known diversity in bats of the extensively analyzed *Lyssavirus* genus, the diversity of other *Rhabdoviridae* genera in bats remains largely undetermined. Vesiculoviruses (genus *Vesiculovirus*), such as vesicular stomatitis virus, cause fever and vesicular diseases in animals such as cattle, horses, and pigs. Some vesiculoviruses, including Chandipura virus and vesicular stomatitis virus, are also zoonotic and cause acute diseases in humans.

**Figure:**
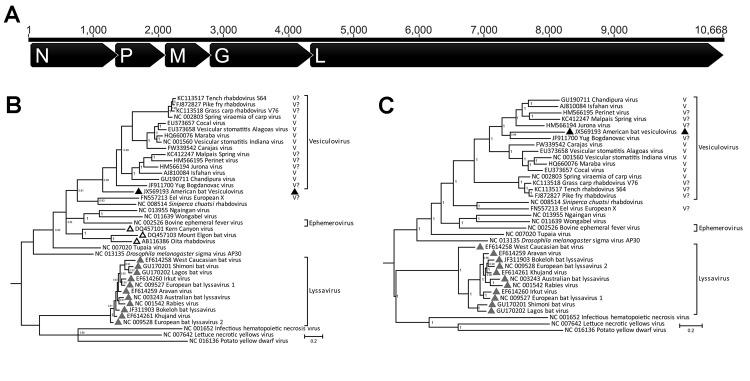
Analyses of American bat vesiculovirus (ABVV) compared with other members of the family *Rhabdoviridae*. A) Genome organization of ABVV; B) Bayesian inference tree of the ABVV N gene; C) Bayesian inference tree of the 5 concatenated ABVV genes (N, P, M, G, L). For the Bayesian analyses, sequences from the entire gene were used, except for a few partially sequenced genomes for which only ≈100 aa were publicly available. Posterior probabilities (>75%) of the Bayesian analysis are shown next to each node. Formally classified vesiculoviruses are labeled with “V,” whereas potential vesiculoviruses not formally recognized by the International Committee on Taxonomy of Viruses are labeled with “V?.” Distinct clades of bat rhabdoviruses are labeled with triangles of different colors: black, vesiculovirus; gray, lyssavirus; white, unclassified. N, nucleoprotein; P, phosphoprotein; M, matrix protein; G, glycoprotein; L, polymerase protein. Scale bar indicates amino acid substitutions per site.

The bat virome has not been fully characterized. Most bat virome studies have been conducted by analyzing fecal, anal swab, or pharyngeal swab specimens from healthy bats (*10*–*13*). These studies have revealed a variety of viruses but no new rhabdoviruses. However, viruses in fecal and pharyngeal samples could include ingested and inhaled viruses that originated from insects and plants (*10*–*13*). To focus specifically on viruses infecting the bats themselves, we performed unbiased metagenomic sequencing of RNA viruses purified from the lungs and livers of 120 rabies-negative big brown bats (*Eptesicus fuscus*) collected in Maryland, USA.

## The Study

During 2008, more than 500 bats associated with possible human exposure were submitted to the Maryland Department of Health and Mental Hygiene State Laboratory for postmortem diagnosis of rabies by direct fluorescent antibody assay (Technical Appendix). For this study, virus particles were purified from the lungs and livers of 120 rabies-negative bats with good carcass condition, and viral nucleic acids were extracted, randomly amplified, and sequenced by using 454 pyrosequencing (Roche, Mannheim, Germany) and Solexa Illumina sequencing (Illumina, San Diego, CA, USA). A total of ≈100,000 pyrosequences and 13.5 million Solexa Illumina sequences were generated and then assembled to form contigs. More than 30 contigs showed low protein identities to known vesiculoviruses (BLASTx; http://blast.ncbi.nlm.nih.gov/Blast.cgi), indicating a possible novel virus. PCR and rapid amplification of cDNA ends were performed to obtain the complete genome of this virus (primers shown in Technical Appendix Figures 1, 2). We proposed the name American bat vesiculovirus (ABVV) for this virus. 

The full-length ABVV genome (GenBank accession no. JX569193) consists of 10,692 nt of negative-sense, single-stranded RNA beginning with a 103-nt 5′ untranslated region, followed by open reading frames encoding for the nucleoprotein, phosphoprotein, matrix protein, glycoprotein, and polymerase protein genes (Figure, panel A). Phylogenetic analyses showed that ABVV is related to vesiculoviruses including Chandipura virus and Isfahan virus, both of which are associated with encephalitic illness in humans. ABVV is located close to the root of vesiculoviruses in the Bayesian analysis of the nucleoprotein gene (Figure, panel B) and shares 41%–49% aa identity with known vesiculoviruses, similar to the vesiculovirus interspecies identities reported (47.9%–72.5%) and higher than the intergenera identities between vesiculoviruses, lyssaviruses, and ephemeroviruses (17.0%–33.1%) (*14*). Analyses of the polymerase gene alone (Technical Appendix Figure 3) and of a concatenation of all 5 genes (Figure, panel C) suggested that ABVV lies within the vesiculovirus clade, more closely related to mammalian than fish vesiculoviruses. Combined, these analyses indicate that ABVV is likely to belong to the *Vesiculovirus* genus, rather than representing a novel genus. The basal phylogenetic position of ABVV suggests early divergence from other mammalian vesiculovirus species.

Lung and liver tissues from 60 of the bats used for the pooled metagenomic analyses were screened individually for ABVV by using reverse transcription PCR targeting the polymerase gene (Technical Appendix). Three (5%) bats tested positive for ABVV: 1 adult female, 1 adult male, and 1 juvenile male. Viral RNA was found in liver tissue from the 2 male bats and in lung and liver tissues from the female adult bat.

Considering the extensive lyssavirus diversity in bats, we hypothesize that bat vesiculoviruses are similarly diverse. To facilitate characterization of diverse vesiculoviruses in bats, we designed 2 pairs of degenerate PCR primers (VesiConAF-KCDGAYAARAGYCAYTCVATGA; VesiConAR-TGNGCNACDGTNARDGCATT; VesiConBF-GGNMGRTTYTTYTCHYTDATGTC; VesiConBR-TCHGCNGAYTGCATNGTYTCA) on the basis of a sequence alignment of the polymerase gene of ABVV and the formally classified mammalian vesiculoviruses. When the ABVV-positive bat liver cDNA was used as a control, the nested PCR yielded an amplicon of 704 bases, and its sequence was confirmed by cloning and Sanger sequencing. Future studies may use these pan-vesiculovirus PCR primers to investigate vesiculovirus diversity in other bat species and in other regions.

## Conclusions

Big brown bats are prevalent in North America, where their geographic range overlaps extensively with that of humans, and considerable interactions occur between big brown bats and humans and their pets. Big brown bats from this region are a known reservoir of rabies virus; our analysis shows that these bats also constitute a sylvatic mammalian reservoir of vesiculoviruses.

The characterization of ABVV sheds light on vesiculovirus diversity in bats. The other bat rhabdoviruses—Mount Elgon bat virus, Oita rhabdovirus, and Kern Canyon virus—do not belong to the vesiculovirus clade but cluster together in a separate clade (*14*) (Figure, panel B). A recent report described several rhabdoviruses in oropharyngeal swab specimens from Spanish bats, but the short reads (100 bases) precluded a detailed phylogenetic analysis (*15*). Of the bats tested in our study, 5% were infected with ABVV, a finding that suggests vesiculoviruses are prevalent in bats. The characterization of a novel rhabdovirus in bats with a history of human contact raises questions for further research, including health effects on the virus’ hosts, seroprevalence, possible transmission by insect vectors, and the extent of zoonotic exposure in humans. ABVV-specific and vesiculovirus-consensus PCRs, as well as future endeavors to culture this virus, will help address these questions.

Technical Appendix Detailed methods for postmortem diagnosis of rabies by direct fluorescent antibody assay in >500 bats associated with possible human exposure submitted to the Maryland Department of Health and Mental Hygiene State Laboratory; location of the primers used in this study relative to the American bat vesiculovirus; sequences of the primers used in this study; and Bayesian inference tree based on the polymerase gene depicting relationships among the members of the family *Rhabdoviridae*.
