# Supplementary material for: Distinct Lineage of Vesiculovirus from Big Brown Bats, United States
Source: Emerg Infect Dis. 2013 Dec;19(12):1978–80. doi: 10.3201/eid1912.121506 (PMC3840891; doi:10.3201/eid1912.121506)
Supplement: Technical Appendix — Detailed methods for postmortem diagnosis of rabies by direct fluorescent antibody assay in >500 bats associated with possible human exposure submitted to the Maryland Department of Health and Mental Hygiene State Laboratory; location of the primers used in this study relative to the American bat vesiculovirus; sequences of the primers used in this study; and Bayesian inference tree based on the polymerase gene depicting relationships among the members of the family Rhabdoviridae. [file 12-1506-Techapp-s1.pdf]

Article DOI: http://dx.doi.org/10.3201/eid1912.121506

Distinct Lineage of Vesiculovirus from Big Brown Bats, United States

Technical Appendix

Materials and Methods

Sample collection and viral metagenomic sequencing

In 2008, >500 bats associated with possible human exposure were submitted to the Maryland Department of Health and Mental Hygiene State Laboratory for the postmortem diagnosis of rabies by direct fluorescent antibody assay. The carcasses of the bats negative for rabies were kept frozen at –80°C. A total of 120 bats were selected for necropsy, including 30 individuals for each of the following categories: juvenile male, juvenile female, adult male and adult female. The lungs and livers were dissected from the bats and pooled for virus purification and metagenomic sequencing according to previously described protocols (*1*,*2*). In short, virus particles were purified from the tissues using homogenization, filtration and nuclease treatment. Purified viral RNA was amplified with random primers using the TransPlex Whole Transcriptome Amplification kit (Sigma-Aldrich) according to manufacturer's instructions. The resulting shotgun libraries were sequenced using 454 pyrosequencing with GS FLX+ Titanium, as well as Solexa Illumina sequencing. A total of 100 thousand pyrosequences and 13.5 million Illumina sequences were generated. Sequences were trimmed and those sharing at least 95% nucleotide identities over 35 bases were assembled into contigs (*3*). Assembled contigs were compared to the GenBank non-redundant protein database using BLASTx with an E-value cutoff of 10^{-4} (*4*).

Complete genome sequencing

The complete genome of the American bat vesiculovirus (ABVV) was obtained by Sanger dideoxy sequencing of PCR products obtained using combinations of primers designed throughout the genome (Technical Appendix Figures 1 and 2) and the 5′ and 3′ extremities were obtained using Rapid Amplification of cDNA Ends (RACE). PCR primers were designed from metagenomic contigs using PRIMER3 (*5*). PCRs were performed using LA Taq (Clontech) with reagent concentrations according to the manufacturer's instructions. PCR reactions were carried out with a "universal touch-down PCR" suitable for the melting temperatures of all primers, as follows: 95°C for 5 min, 45 cycles of [94°C for 1 min, 58°C minus 0.2°C per cycle for 1 min, 72°C for 1 to 5 min], followed by 72°C for 10 min. Amplicons were sequenced to their entirety by Sanger sequencing. The 5′ and 3′ genome extremities were amplified using RACE amplification kits (Invitrogen) according to the manufacturer's instructions and previously described protocols (*4*).

To determine the relationship of ABVV to other rhabdoviruses, a phylogram was created based on the amino acid sequence encoded by the nucleoprotein (N), polymerase (L) gene, as well as the five gene concatenated alignment. The deduced amino acid sequences were aligned using Mafft 5.8 (6) with the E-INS-I alignment strategy and previously described parameters (*4*,*7*). Bayesian inference trees were constructed using MrBayes (*8*). The Markov chain was run for a maximum of 1 million generations, with a stopping rule implemented so that the analysis would halt when the average deviation of the split frequencies was less than 0.01%. Every 50 generations were sampled and the first 25% of mcmc samples were discarded as burn-in. Mid-point rooting was conducted using MEGA (*9*).

Specific reverse transcription PCR (RT-PCR) for ABVV and consensus RT-PCR for mammalian vesiculoviruses

RNA was extracted from the liver and lung tissues of 60 bats using the QIAamp MinElute Virus Spin kit (Qiagen). cDNA was generated from the sample RNA using the SuperScript III reverse transcription (RT; Invitrogen) with 100 pmol of random hexamer primer, 10 pmol of each dNTP, 10 µL of RNA, 1 µL buffer, 5mM DTT, 1 µL of RiboLock RNase Inhibitor (Fermentas), and 200 units of RT enzyme following the manufacturer’s instruction. PCR primers ABVV-AF (5′^{6333} CGACCTGATGAGAGTGGTGA 3′) and ABVV-AR (5′ ^{6795}AGTCGGGAGTTGATCATTGG 3′) were used in PCR reactions targeting the polymerase gene of ABVV, producing an amplicon of 463 nt. The PCR reaction (containing 1 µM of each primer, 200 µM dNTPs, 1 U RedTaq DNA Polymerase (Sigma-Aldrich), 1X Red Taq Reaction Buffer, and 5 µl of target DNA in a 50 µl reaction) was carried out with the touch-down PCR conditions described above for genome completion. Amplicons were analyzed by ethidium bromide gel electrophoresis.

For the mammalian-vesiculovirus-consensus PCR, two pairs of degenerate PCR primers were designed based on the sequence alignment of the polymerase gene of formally classified mammalian vesiculoviruses (Figure 1 in main text; Technical Appendix Figure 3). Primers VesiConAF (5′KCDGAYAARAGYCAYTCVATGA 3′) and VesiConAR (5′ TGNGCNACDGTNARDGCATT 3′) were used for the first round of PCR. VesiConBF (5′ GGNMGRTTYTTYTCHYTDATGTC 3′) and VesiConBR (5′ TCHGCNGAYTGCATNGTYTCA 3′) were used for the second round of PCR with 2.5 µl of the first round PCR product The LA Taq reaction composition and touch-down PCR was performed as described above.

References

1. Ng TFF, Manire C, Borrowman K, Langer T, Ehrhart L, Breitbart M. Discovery of a novel single-stranded DNA virus from a sea turtle fibropapilloma by using viral metagenomics. J Virol. 2009;83:2500–9. PubMed http://dx.doi.org/10.1128/JVI.01946-08

2. Ng TFF, Willner DL, Lim YW, Schmieder R, Chau B, Nilsson C, et al. Broad surveys of DNA viral diversity obtained through viral metagenomics of mosquitoes. PLoS ONE. 2011;6:e20579. PubMed http://dx.doi.org/10.1371/journal.pone.0020579

3. Schmieder R, Edwards R. Quality control and preprocessing of metagenomic datasets. Bioinformatics. 2011;27:863–4. PubMed http://dx.doi.org/10.1093/bioinformatics/btr026

4. Ng TFF, Marine R, Wang C, Simmonds P, Kapusinszky B, Bodhidatta L, et al. High variety of known and new RNA and DNA viruses of diverse origins in untreated sewage. J Virol. 2012;86:12161–75. PubMed http://dx.doi.org/10.1128/JVI.00869-12

5. Rozen S, Skaletsky H. Primer3 on the WWW for General Users and for Biologist Programmers. Bioinformatics Methods and Protocols; 1999. p. 365–86.

6. Katoh K, Kuma K-i, Toh H, Miyata T. MAFFT version 5: improvement in accuracy of multiple sequence alignment. Nucleic Acids Res. 2005;33:511–8. PubMed http://dx.doi.org/10.1093/nar/gki198

7. Ng TFF, Wheeler E, Greig D, Waltzek TB, Gulland F, Breitbart M. Metagenomic identification of a novel anellovirus in Pacific harbor seal (*Phoca vitulina* richardsii) lung samples and its detection in samples from multiple years. J Gen Virol. 2011;92:1318–23. PubMed http://dx.doi.org/10.1099/vir.0.029678-0

8. Huelsenbeck JP, Ronquist F. MRBAYES: Bayesian inference of phylogenetic trees. Bioinformatics. 2001;17:754–5. PubMed http://dx.doi.org/10.1093/bioinformatics/17.8.754

9. Tamura K, Dudley J, Nei M, Kumar S. MEGA4: Molecular evolutionary genetics analysis (MEGA) software version 4.0. Mol Biol Evol. 2007;24:1596–9. PubMed http://dx.doi.org/10.1093/molbev/msm092

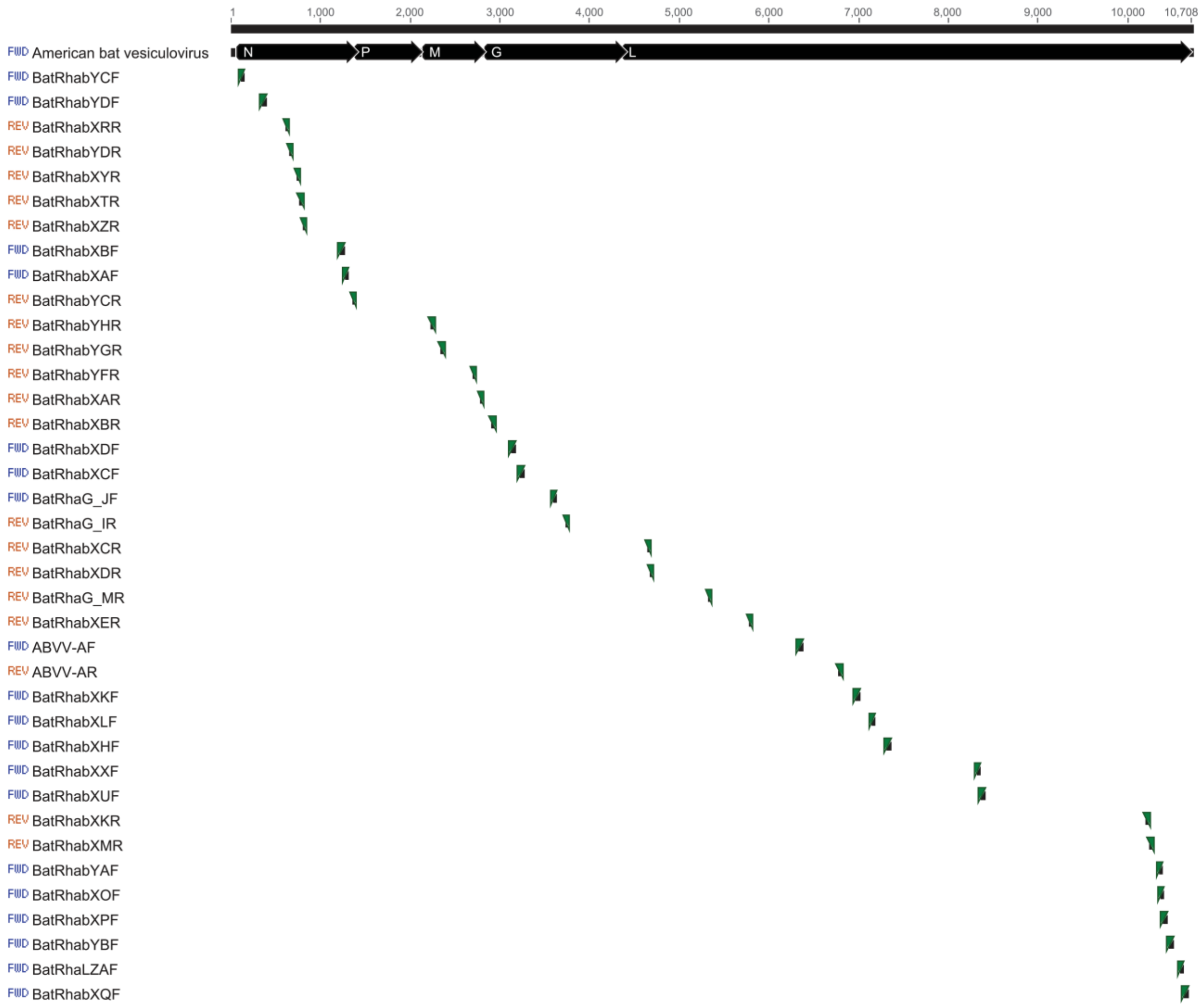

Technical Appendix Figure 1. Location of the primers used in this study relative to the American bat vesiculovirus.

Primer	Sequence (positions 1–27)
BatRhabYCF	TCTCGCGTAAGAGACGTTGA
BatRhabYDF	AGACCAACGGTGAGAAATGG
BatRhabXRR	GAGCGACAGCTCTATGAGAAAGA
BatRhabYDR	TTTCCCCATCCGTCATAAAA
BatRhabXYR	CAAATCGAATGACTGCCCGGTCTGA
BatRhabXTR	GCTGCACAATCCTTGTATCG
BatRhabXZR	CCGGTGACCTTGTTGAGATGGGCAAA
BatRhabXBF	GCAGAGACTGTGCAGAATGG
BatRhabXAF	GTATCCCGACTGCCGATATG
BatRhabYCR	CCCTCAAAATAAGCCTTAGCAA
BatRhabYHR	CAACTCCCGGGGCTCCTTTGGTCTC
BatRhabYGR	CGAAACAATCCTGAGGGATCCCCGAAA
BatRhabYFR	TGGACGGGTTGAGGATTTGATCCAAGG
BatRhabXAR	TAGCTTGTCGCCATCTTCCT
BatRhabXBR	TGACCGGCAGTATGACTTTG
BatRhabXDF	AAAGAGTCCGCCACAGTGTT
BatRhabXCF	GGATTTCCACCACCATCTTG
BatRhaG_JF	TCCAGGAGGATCTTGGTCAT
BatRhaG_IR	CCTCTCCAAGTCCCATACCA
BatRhabXCR	TCAGCAGATTTGTGCCAGTC
BatRhabXDR	TGATCCAAGACCCAAACCAT
BatRhaG_MR	TTCTTGACGTGCTCTCCAAA
BatRhabXER	CGGTTCATTGAATGGCTCTT
ABVV-AF	CGACCTGATGAGAGTGGTGA
ABVV-AR	AGTCGGGAGTTGATCATTGG
BatRhabXKF	GACAATTTGGCGTTCAAGGT
BatRhabXLF	CAATTGTGCGTTTCTTTTGG
BatRhabXHF	GATCGTCCGAGATGCAGTTA
BatRhabXXF	TGTGCGAGTCGATCACATCT
BatRhabXUF	CTTGGGGAGACCCTGTTGTA
BatRhabXKR	GCCTGGTCTTTTGGAGAACA
BatRhabXMR	CAGAGCACTAGCCATTCTCG
BatRhabYAF	CTTCCCCATCCGAATTCGCGTGACA
BatRhabXOF	TTCGCGTGACAAAAGTGAAC
BatRhabXPF	TCATGGAGTTGTCACGGAGA
BatRhabYBF	TCGGGCAATGGATTAGGACCCTACACA
BatRhaLZAF	CGACTTAACAGGGATAAAGGATG
BatRhabXQF	ACAAGTCTGTGCCGTTTGCT
VesiConAF	KCDGAYAARAGYCAYTCVATGA
VesiConAR	TGNGCNACDGTNARDGCATT
VesiConBF	GGNMGRTTYTTYTCHYTDATGTC
VesiConBR	TCHGCNGAYTGCATNGTYTCA

Technical Appendix Figure 2. Sequences of the primers used in this study. IUPAC nucleotide ambiguity codes were used for the degenerate primers.

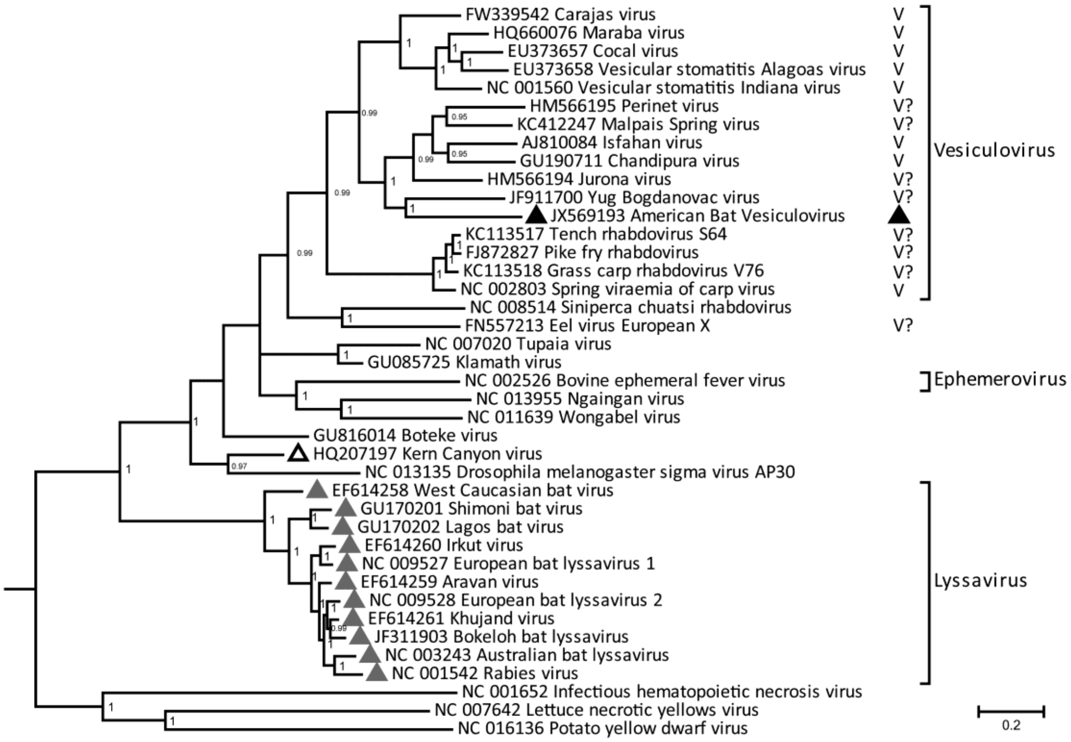

Technical Appendix Figure 3. Bayesian inference tree based on the polymerase (L) gene depicting relationships among the members of the family *Rhabdoviridae*.
